# Elevated Serum Concentration of Adipocyte Fatty Acid-Binding Protein Correlates with the Markers of Abdominal Obesity Independently of Thyroid Hormones in Non-Obese Women with Polycystic Ovary Syndrome

**DOI:** 10.3390/jcm12144610

**Published:** 2023-07-11

**Authors:** Aleksandra Maria Polak, Agnieszka Łebkowska, Anna Krentowska, Angelika Buczyńska, Marcin Adamski, Adam Jacek Krętowski, Irina Kowalska, Agnieszka Adamska

**Affiliations:** 1 Department of Endocrinology, Diabetology and Internal Medicine, Medical University of Białystok, 15-276 Białystok, Poland; 2 Department of Internal Medicine and Metabolic Diseases, Medical University of Białystok, 15-276 Białystok, Poland; 3 Clinical Research Centre, Medical University of Białystok, 15-276 Białystok, Poland; 4 Faculty of Computer Science, Bialystok University of Technology, 15-351 Białystok, Poland

**Keywords:** A-FABP, thyroid hormones, obesity, PCOS

## Abstract

Adipocyte fatty acid-binding protein (A-FABP) is mainly expressed in adipocytes. The risk of abdominal obesity and autoimmune thyroid disease is increased in women with polycystic ovary syndrome (PCOS). The objective of this study was to explore the relationship of serum concentration of A-FABP with parameters of obesity, e.g., waist to hip ratio (WHR) and the amount of adipose tissue assessed by bioelectrical impedance analysis (BIA), and thyroid hormone homeostasis in women with PCOS. We examined 66 women with PCOS and 67 healthy women. Serum concentrations of A-FABP and thyroid hormones were measured; the FT3/FT4 ratio, thyroid-stimulating hormone index (TSHI), thyrotrope thyroxine resistance index (TT4RI) and thyroid feedback quantile-based index (TFQI) were calculated. In the PCOS group, serum concentrations of A-FABP, FT3 and the FT3/FT4 ratio were significantly higher in comparison to the control group (all *p* < 0.05). A correlation of A-FABP with WHR (r = 0.26, *p* = 0.04) and the percentage of adipose tissue (r = 0.33, *p* = 0.01) has been found only in women with PCOS. We observed no correlation between serum levels of A-FABP and TSHI, TT4RI or TFQI in women with PCOS (all *p* > 0.05). Our results indicate that A-FABP is an adipokine that may be connected with abdominal obesity independently of thyroid hormone homeostasis in PCOS patients.

## 1. Introduction

Polycystic ovary syndrome (PCOS) is considered the most common endocrinopathy, affecting up to 20% of women in reproductive age [1]. According to Rotterdam criteria, PCOS is diagnosed when at least two of the following features are present: clinical and/or biochemical hyperandrogenism, oligoovulation and/or anovulation and polycystic ovarian morphology assessed in transvaginal ultrasound [2]. Insulin resistance and consequent hyperinsulinemia are important factors in PCOS pathogenesis and are associated with an increased risk of metabolic syndrome, obesity and type 2 diabetes [3]. It has been shown that overweight or obesity are present in more than 50% of PCOS patients [4].

Adipose tissue is considered the primary storage of excess energy, but also an active endocrine organ. Adipocytes synthetize and release a number of biologically active substances, including adipocyte fatty acid-binding protein (A-FABP) [5], which is expressed in mature cells of adipose tissue, participates in the transport of fatty acids to cell compartments and modulates intracellular lipid metabolism [6]. It has been reported that increased serum A-FABP concentration is associated with a decrease in insulin sensitivity [7]. Moreover, circulating A-FABP positively correlates with the components of metabolic syndrome [7], as well as the occurrence of atherosclerosis and cardiovascular diseases [8,9,10]. Animal models have shown that mice deficient in A-FABP were protected from the development of insulin resistance, impaired glucose tolerance and atherosclerosis [11,12,13]. In a previous study, a significantly higher serum concentration of A-FABP in women with PCOS compared to the control group has been revealed [14]. Moreover, the authors found a significantly higher concentration of A-FABP in obese patients and a positive correlation of body mass index (BMI) and HOMA-IR with A-FABP. Similarly, Mohling et al. showed that serum A-FABP concentration correlated significantly with BMI and the amount of adipose tissue assessed using dual energy X-ray absorptiometry (DEXA) method, although they did not find a significant relationship between A-FABP concentration and insulin resistance [15].

The central sensitivity to thyroid hormones, which reflects the grade of pituitary gland inhibition by FT4 concentration, is determined on the basis of the interaction between FT4 and TSH [16]. Thus, thyroid-stimulating hormone index (TSHI), thyrotrope thyroxine resistance index (TT4RI) and thyroid feedback quantile-based index (TFQI) are considered central indices of thyroid hormone sensitivity [16,17]. In peripheral tissues, FT4 is converted to FT3 by iodothyronine deiodinase (DIO), and the FT3/FT4 ratio is therefore considered to reflect peripheral sensitivity to thyroid hormones [17]. It has been found that the FT3/FT4 ratio is positively associated with both waist circumference and BMI [18]. Posadas-Romero et al. found a positive association of subclinical hypothyroidism with insulin resistance, obesity and metabolic syndrome [19]. Moreover, Ambrosi et al. revealed a positive relationship of serum TSH concentrations with BMI [20]. Metabolic effects of thyroid hormones (FT3, FT4) and TSH are closely related to the energy metabolism of the body, and therefore to the mechanisms regulating body weight balance through stimulation of thermogenesis and lipid catabolism [21]. One of the main target tissues for thyroid hormones are adipocytes. Moreover, preadipocytes and adipocytes have receptors for TSH, and this signaling pathway is involved in the processes of adipogenesis, which suggests, in addition to the abovementioned mechanism, that the alteration of TSH concentration itself may promote obesity [22]. Previous reports suggest that decreased sensitivity to thyroid hormones is linked to metabolic disorders [16]. Nannipieri et al. have shown a decrease in expression of the thyroid hormone receptor (THR) and TSH receptor in subcutaneous adipose tissue of obese subjects compared to controls [23].

Some studies suggest that A-FABP may constitute a link between obesity and thyroid hormone homeostasis. Nie et al. have observed that increased serum A-FABP levels are associated with decreased sensitivity to thyroid hormones in the euthyroid population [17]. Considering the insufficient data, especially in PCOS patients, the purpose of this study was to assess the relationships of A-FABP with parameters of obesity and indices of thyroid hormone homeostasis in women with PCOS.

## 2. Materials and Methods

### 2.1. Subjects

The study population consisted of 133 women: 66 patients with PCOS and 67 healthy women with comparable BMI. PCOS women were patients of the Department of Endocrinology, Diabetology and Internal Medicine and the Department of Internal Medicine and Metabolic Diseases, Medical University of Białystok. The control group was recruited among students who met the following inclusion criteria: no signs of clinical or biochemical hyperandrogenism; regular, ovulatory menstrual cycles; and a normal ovarian morphology assessed in transvaginal ultrasound. PCOS was diagnosed using the 2003 Rotterdam European Society of Human Reproduction and Embryology/American Society of Reproductive Medicine (ESHRE/ASRM) PCOS Consensus Workshop Group diagnostic criteria, i.e., the presence of ≥2 of the following criteria: clinical and/or biochemical hyperandrogenism, oligo/anovulation and polycystic ovaries on ultrasound (≥12 follicles measuring 2–9 mm in diameter or ovarian volume >10 mL in at least one ovary) [2]. The exclusion criteria for both groups included other causes of irregular menstrual cycles and/or hyperandrogenism, i.e., hyperprolactinemia; Cushing’s syndrome (excluded with history taking and physical examination); late-onset congenital adrenal hyperplasia (excluded with serum concentrations of 17-hydroxyprogesterone); hypo- or hyperthyroidism; pregnancy (excluded with an appropriate test) and/or breastfeeding; type 1 or type 2 diabetes; chronic or acute infection (in the previous month); any other serious medical condition; hormonal contraception and/or anti-androgen therapy (in the previous 6 months); and current pharmacological treatment of obesity, hyperglycemia, dyslipidemia or hypertension.

The study protocol was approved by the Ethics Committee of the Medical University of Białystok, Poland (approval no APK.002.476.2021) and was concordant with the Declaration of Helsinki. All subjects signed an informed consent form after obtaining full information on the purpose of the study and the performed procedures.

### 2.2. Anthropometric Measurements

All women underwent physical examination. BMI was calculated as body weight [kg]/height [m^2^]. Waist circumference was measured at the smallest circumference between the rib cage and the iliac crest, while hip circumference was measured at the maximum circumference at the level of the femoral trochanters, both in the standing position. Waist–hip ratio (WHR) was calculated as waist circumference [cm]/hip circumference [cm].

### 2.3. Biochemical Analyses

In all women, fasting blood samples were collected between the 3rd and 6th day of the menstrual cycle or at least 3 months from the last menses, independently of cycle phase, in patients with amenorrhea. Moreover, an oral glucose tolerance test with 75 g with the determination of plasma glucose and serum insulin concentrations at 0 and 120 min was performed. Plasma glucose and serum insulin concentrations, levels of serum luteinizing hormone (LH) and follicle-stimulating hormone (FSH), total testosterone, serum sex hormone-binding globulin (SHBG), plasma total cholesterol (TC), high-density lipoprotein cholesterol (HDL-C), triglycerides (TG) and low-density lipoprotein cholesterol (LDL-C) were assayed as previously described [24]. Serum concentrations of TSH, FT3 and FT4 were measured by electrochemical luminescence immunoassay as previously described [25].

The quantitative determination of serum A-FABP concentrations was performed using an enzyme-linked immunosorbent assay (ELISA) kit characterized by a sensitivity equal to 0.059 ng/mL; intra-assay coefficient of variation (CV)—<10%; and inter-assay CV—<12% (Cloud-Clone Corp., Wuhan, Hubei 430056, China, SEB693Hu).

### 2.4. Calculations

FAI was calculated as total testosterone [nmol/L] × 100/SHBG [nmol/L] [26].

Insulin resistance was assessed with the homeostasis model assessment index (HOMA-IR), calculated with the formula (fasting insulin [µU/mL] × fasting glucose [mmol/L])/22.5 [27].

Peripheral sensitivity to thyroid hormones was assessed with the FT3 to FT4 ratio (FT3/FT4). Central sensitivity to thyroid hormones was evaluated by the calculation of TSHI, TT4RI and TFQI as previously described [16,17].

### 2.5. Body Composition Analysis

Body composition was assessed using bioelectrical impedance method (InBody720, Inbody Co., Ltd., Seoul, Republic of Korea) at the Clinical Research Centre, Medical University of Białystok by qualified physicians. The equipment was calibrated before every examination. The patients were positioned on the examination platform in a standing position with feet wide apart and straight arms at a short distance from the body. The duration of each examination was approximately 2 min. Based on the assessment of the electrical resistance of tissues, the percentage of adipose tissue was estimated.

### 2.6. Statistical Analysis

Statistical analyses were performed using the Statistica 13.3 package (Statsoft, Cracow, Poland). The distribution of variables was evaluated with the Kolmogorov–Smirnov test, which showed a non-normal distribution of data. Because of that, non-parametric tests were used, and all values of continuous variables were expressed as the median (interquartile range). The comparisons between the study groups were performed with the Mann–Whitney U test. Correlation analysis was performed with the Spearman test. A *p*-value < 0.05 was considered statistically significant.

## 3. Results

The clinical and biochemical characteristics of the studied groups are presented in Table 1.

The studied groups were comparable in terms of age, BMI, WHR and percentage of adipose tissue (all *p* > 0.05). As expected, total testosterone concentration and FAI were significantly higher in women with PCOS in comparison to healthy women (both *p* < 0.05), while the concentrations of SHBG did not differ. No differences regarding serum concentrations of LH, FSH, lipids, glucose during OGTT, fasting insulin or HOMA-IR between PCOS and control groups were observed (all *p* > 0.05), although insulin concentration at 120 min of OGTT was higher in women with PCOS.

No significant differences in serum concentration of TSH or FT4 between the studied groups were demonstrated (all *p* > 0.05), although women with PCOS had a significantly higher serum concentration of FT3 in comparison to the control group (*p* < 0.01). Additionally, our studied groups did not differ in values of TFQI, TT4RI and TSHI (all *p* > 0.05), whereas the FT3/FT4 ratio was significantly higher in PCOS women (*p* = 0.02) (Table 1). A higher serum level of FABP4 was found in patients with PCOS in comparison to the control group (*p* = 0.04).

We did not find any correlation between A-FABP and glucose, insulin or HOMA-IR in the studied groups (all *p* > 0.05).

In the PCOS group, we observed correlations of serum A-FABP concentration with WHR (r = 0.26, *p* = 0.04) and percentage of adipose tissue assessed by bioelectrical impedance analysis (r = 0.33, *p* < 0.01), whereas these correlations were not found in the control group (all *p* > 0.05). We observed no association of serum concentration of A-FABP with BMI, total testosterone, FAI or SHBG in the PCOS patients or in the control group (all *p* > 0.05). In the PCOS women, as well as in the controls, we did not observe an association of serum concentration of A-FABP with TSH, FT3 or FT4 (Table 2).

In our study, we did not demonstrate any association of A-FABP with the FT3/FT4 ratio in the studied groups (all *p* > 0.05). No associations between serum levels of A-FABP and TSHI, TT4RI or TFQI were observed in women with PCOS (all *p* > 0.05). In the control group, we found positive associations of A-FABP with TT4RI (r = 0.28, *p* = 0.02) and TSHI (r = 0.28, *p* = 0.02) (Table 3).

## 4. Discussion

The present study demonstrates that women with PCOS are characterized by an increased serum level of A-FABP in comparison to healthy women. These results are consistent with the findings of previously published reports [14]. In a study comparing gene expression profiles between patients with PCOS and control women, it has been shown that A-FABP mRNA expression is significantly increased in PCOS [14]. Accordingly, Hu et al. [14] have reported that serum A-FABP concentrations were higher in obese and non-obese PCOS women than in obese and non-obese controls, respectively. Moreover, they found that in the PCOS group, A-FABP concentrations were positively correlated with serum total testosterone. On the other hand, Mohlig et al. [15] and Lázaro et al. [28] found no association between A-FABP and serum testosterone concentration. In our study, PCOS women were shown to have a higher serum concentration of total testosterone and FAI compared with healthy women. In PCOS women, obesity is tightly associated with hyperandrogenemia [29]. Therefore, we investigated an association of serum concentration of A-FABP with serum testosterone concentration and FAI, though we did not find an association. Thus, the obtained data cannot support the hypothesis that circulating A-FABP links obesity with hyperandrogenemia in PCOS women.

Previously published studies have shown that an increased serum concentration of A-FABP is associated with a decrease in insulin sensitivity [7,30,31]; however, in our study, we found no relation between A-FABP and HOMA-IR. There is increasing evidence indicating the relationship of metabolic syndrome and obesity with serum A-FABP concentrations [7,32]. However, we did not observe an association of A-FABP with BMI, while other researchers suggest that A-FABP correlates positively with metabolic syndrome [28], as well as with BMI [33]. A discrepancy in the results may arise from the fact that the previous reports focused primarily on the relationship between the serum concentration of A-FABP and BMI [34]. However, although BMI is a widely used anthropometric indicator of obesity, it does not provide information on fat mass (FM) and fat-free mass (FFM). In our study, we found an association of A-FABP with WHR only in women with PCOS. Moreover, apart from BMI and WHR, we used bioelectrical impedance analysis to accurately measure total body fat. To date, the association between serum concentrations of A-FABP and total body fat assessed with bioelectrical impedance has been the subject of a few studies [7,30]. Consistent with their findings, our study demonstrated a positive association of A-FABP with the percentage of adipose tissue assessed by bioelectrical impedance in PCOS patients, whereas in the control group no such associations were found. As it was mentioned above, the main finding of the present study is a higher serum A-FABP concentration in non-obese women with PCOS compared to age- and BMI-matched control women. In the PCOS group, A-FABP positively correlated with WHR and the percentage of adipose tissue assessed by BIA, though PCOS women did not present a higher amount of adipose tissue. This evidence, together with the lack of differences in percentage of adipose tissue between both studied groups, suggests that some metabolic abnormalities in the functioning of visceral adipose tissue exist in non-obese PCOS patients and that this phenomenon is transient. The achievement of this study seems to be the demonstration that non-obese PCOS women present metabolic abnormalities even before the onset of obesity. During unfavorable adipose tissue homeostasis, as an adaptive response to the body’s energetic demand, preadipocytes and adipocytes may show significant expansion through the proliferation of tissue-resident precursors and differentiation in mature adipocytes (hyperplasia) as well as an increase in cell size of existing adipocytes (hypertrophy) [35]. These processes of adipose tissue remodeling leading to subsequent adipocyte dysfunction may mean further susceptibility to obesity and obesity-related complications. Our previous study [36] may support the abovementioned hypothesis of abnormalities in the functioning of visceral adipose tissue in PCOS women even with the correct amount of adipose tissue. We did not find differences in serum leptin concentrations between PCOS and the control group, which may be explained by the lack of differences in BMI between the studied groups. This statement is based on the fact that leptin, which is considered as an adipokine closely related to obesity, is produced proportionally to the amount of adipose tissue [37]. Moreover, in a state of the same macronutrient intake in both studied groups, we found a positive association of dietary fat and saturated fatty acids intake with serum leptin concentrations exclusively in PCOS women. Additionally, we observed a positive association between dietary fat intake and HOMA-IR, whereas these correlations were not observed in the control group.

It should be emphasized that this study was focused only on non-obese patients. In the case of obesity-related PCOS, there might be different pathways that influence PCOS risk. The Rotterdam criteria for PCOS recognize four clinical phenotypes of the syndrome. The most prevalent phenotype is the classic form [38], which meets all three current criteria for PCOS: clinical and/or biochemical hyperandrogenism (HA), menstrual dysfunction (oligo/amenorrhea) (Oligo) and polycystic ovarian morphology (PCOM)-phenotype A (Oligo + HA + PCOM). Phenotype B (HA + Oligo) and phenotype C (HA + PCOM) are less frequent. The Rotterdam criteria also recognize a fourth phenotype, D, which is defined by oligomenorrhea, polycystic ovarian morphology in ultrasound and normal androgen levels (Oligo + PCOM) [39]. An increased incidence of metabolic disorders is observed among women with phenotypes A, B and C [40], whereas phenotype D is characterized by fewer metabolic abnormalities [41]. In our previous study [24], it was only in phenotype A that we found a higher visceral adipose tissue mass and android/gynoid ratio (A/G ratio) in comparison to the control group; therefore, metabolic disturbances could be more pronounced in this phenotype. However, not all published data confirm this hypothesis [41].

Previous studies demonstrate an association between the occurrence of obesity and disturbances in thyroid hormone homeostasis [42] and impaired sensitivity to thyroid hormones [16]. In our study, we observed higher serum concentrations of FT3 and the FT3/FT4 ratio in patients with PCOS in comparison to the control group. It has been previously found that the FT3/FT4 ratio, which reflects peripheral sensitivity to thyroid hormones, is positively associated with both waist circumference and BMI [18]. We can speculate that leptin, which is elevated in the serum of PCOS patients [36], may be involved in this process by upregulating the expressions of deiodinases in white adipose tissue and increasing the conversion of thyroxine to triiodothyronine [43]. The present study did not show an association between A-FABP and the FT3/FT4 ratio in the studied groups. Additionally, we did not find differences between PCOS patients and the control group regarding central sensitivity to thyroid hormones which was measured by TSHI, TT4RI and TFQI. We also observed no relation between serum concentrations of A-FABP and TSHI, TT4RI or TFQI in women with PCOS. Previous studies suggest that A-FABP may be a link between obesity and thyroid hormone homeostasis. Nie et al. [17] found a positive association of A-FABP with serum FT4 concentration, although not with FT3 or TSH concentrations. In the present study, no association between the serum levels of A-FABP and TSH, FT4 or FT3 in the analyzed subgroups was found. It has previously been demonstrated that an increased serum A-FABP concentration is associated with lower central and peripheral sensitivity to thyroid hormones [17], though it has not been assessed in PCOS women. In our study, we observed that in the control group there is a positive association of A-FABP with TT4RI and TSHI, suggesting that increased serum A-FABP concentration is associated with a decrease in central sensitivity to thyroid hormones only in the general population. Therefore, we hypothesized that A-FABP is an adipokine which is probably not involved in thyroid hormone homeostasis in PCOS women.

The main limitation of the present study is a relatively small sample size. It would be therefore valuable to perform a study with a larger sample size in the future to validate our results. Moreover, in our study we analyzed non-obese women with PCOS and a control group, so it would be of interest to verify our hypothesis in a group of overweight and obese women. Additionally, this study used BIA for the estimation of body composition, so research using other methods of assessing body composition in this group of patients would be interesting.

## 5. Conclusions

In summary, in our study we observed an association of A-FABP with WHR and the percentage of adipose tissue assessed by BIA, independently of thyroid hormone homeostasis in non-obese PCOS patients. These findings could reflect the transient function of visceral adipose tissue in PCOS patients, because during unfavorable adipose tissue homeostasis preadipocytes and adipocytes may show significant hypertrophy and hyperplasia. The accumulation of hyperplastic cells may mark further susceptibility for obesity and obesity-related complications. Therefore, in this study, PCOS women do not have excess adipose tissue but its function is impaired.

Additionally, we observed higher serum concentrations of FT3 and the FT3/FT4 ratio in patients with PCOS in comparison to the control group. However, we did not notice a relationship between A-FABP and the sensitivity to thyroid hormones. Therefore, we hypothesized that A-FABP is an adipokine which probably is not involved in thyroid hormone homeostasis in PCOS women.

## Figures and Tables

**Table 1:** Clinical and biochemical characteristics of the studied groups.

	Control Group (*n* = 67)	PCOS (*n* = 66)	*p* Value
Age (years)	26 (23.0–28.0)	24.0 (22.0–27.0)	0.06
BMI (kg/m^2^)	22.19 (21.01–24.20)	22.82 (21.4–25.39)	0.37
WHR	0.8 (0.77–0.85)	0.79 (0.76–0.84)	0.61
Percentage of adipose tissue (%)	29.9 (25.2–33.4)	29.6 (24.5–36.4)	0.77
TT (ng/mL)	0.6 (0.45–0.72)	0.71 (0.58–0.88)	<0.01 *
SHBG (nmol/L)	61.74 (43.76–84.57)	52.07 (36.57–66.60)	0.09
FAI	3.04 (1.99–4.54)	4.43 (2.85–6.27)	<0.01 *
FSH (IU/L)	5.43 (4.25–6.58)	5.57 (4.30–6.36)	0.86
LH (IU/L)	3.9 (2.97–5.08)	4.07 (2.98–5.14)	0.61
Total cholesterol (mg/dL)	170.0 (153.0–196.0)	171.5 (159.0–188.0)	0.83
HDL-cholesterol (mg/dL)	64.0 (57.0–78.0)	70.0 (58.0–76.0)	0.58
LDL-cholesterol (mg/dL)	91.2 (72.6–106.8)	90.3 (79.6–103.8)	0.88
TG (mg/dL)	57.0 (40.0–77.0)	59.5 (47.0–76.0)	0.48
Glucose 0′ OGTT (mg/dL)	92.0 (87.0–96.0)	90.5 (87.0–95.0)	0.62
Glucose 120′ OGTT (mg/dL)	90.0 (76.0–101.0)	88.5 (79.0–100.0)	0.89
Insulin 0′ OGTT (uIU/mL)	8.28 (7.25–10.84)	8.63 (6.73–12.07)	0.8
Insulin 120′ OGTT (uIU/mL)	25.46 (17.74–38.42)	30.53 (23.38–51.42)	<0.01 *
HOMA-IR	1.91 (1.63–2.52)	2.0 (1.46–2.80)	0.75
TSH (µIU/mL)	1.79 (1.37–2.48)	1.96 (1.55–2.69)	0.17
FT3 (pmol/L)	3.24 (2.69–3.63)	3.62 (3.27–3.9)	<0.01 *
FT4 (pmol/L)	16.87 (15.39–18.63)	16.6 (15.45–18.74)	0.71
FT3/FT4 ratio	0.3 (0.23–0.37)	0.34 (0.26–0.39)	0.02 *
TFQI	0.07 (−0.35–0.31)	0.01 (−0.23–0.25)	0.71
TT4RI	31.5 (22.9–40.2)	32 (24.5–47.2)	0.53
TSHI	2.9 (2.5–3.2)	2.9 (2.6–3.3)	0.66
A-FABP	1.62 (0.73–3.97)	2.54 (1.15–8.73)	0.04 *

Values are expressed as the median (interquartile range): * *p* < 0.05. Abbreviations: A-FABP: adipocyte fatty acid-binding protein; BMI: body mass index; FAI: free androgen index; FSH: follicle-stimulating hormone; FT3: free triiodothyronine; FT4: free thyroxine; HDL: high-density lipoprotein; HOMA-IR: homeostasis model assessment of insulin resistance; LDL: low-density lipoprotein; LH: luteinizing hormone; OGTT: oral glucose tolerance test; PCOS: polycystic ovary syndrome; SHBG: sex hormone-binding globulin; TFQI: thyroid feedback quantile-based index; TG: triglycerides; TSH: thyroid-stimulating hormone; TSHI: thyroid-stimulating hormone index; TT: total testosterone; TT4RI: thyrotroph thyroxine resistance index; and WHR: waist to hip ratio.

**Table 2:** Correlations of serum A-FABP concentration with anthropometric parameters and hormones in the control and PCOS groups.

	Control Group (*n* = 67)	PCOS (*n* = 66)
BMI	r = 0.11 *p* = 0.36	r = 0.22 *p* = 0.07
WHR	r = −0.06 *p* = 0.64	r = 0.26 *p* = 0.04 *
Percentage of adipose tissue	r = 0.16 *p* = 0.22	r = 0.33 *p* = 0.01 *
TT	r = 0.08 *p* = 0.5	r = −0.09 *p* = 0.48
SHBG	r = 0.002 *p* = 0.99	r = −0.06 *p* = 0.66
FAI	r = 0.08 *p* = 0.53	r = 0.04 *p* = 0.76
TSH	r = 0.22 *p* = 0.07	r = −0.1 *p* = 0.42
FT3	r = 0.19 *p* = 0.13	r = 0.08 *p* = 0.51
FT4	r = 0.09 *p* = 0.5	r = −0.12 *p* = 0.34

Data are derived from Spearman correlation coefficients. The level of significance was accepted at * *p* < 0.05. Abbreviations: BMI: body mass index; FAI: free androgen index; FT3: free triiodothyronine; FT4: free thyroxine; PCOS: polycystic ovary syndrome; SHBG: sex hormone-binding globulin; TSH: thyroid-stimulating hormone; TT: total testosterone; and WHR: waist–hip ratio.

**Table 3:** Correlations between A-FABP and central and peripheral thyroid hormone sensitivity.

	Control Group (*n* = 67)	PCOS (*n* = 66)
FT3/FT4	r = 0.09 *p* = 0.49	r = 0.09 *p* = 0.46
FTQI	r = 0.22 *p* = 0.08	r = −0.12 *p* = 0.33
TT4RI	r = 0.28 *p* = 0.02 *	r = −0.11 *p* = 0.36
TSHI	r = 0.28 *p* = 0.02 *	r = −0.12 *p* = 0.32

The level of significance was accepted at * *p* < 0.05.

## Data Availability

The data presented in this study are available on request from the corresponding author.
